# The effect of an interleukin receptor antagonist (IL-1ra) on colonocyte eicosanoid release

**DOI:** 10.1155/S0962935196000622

**Published:** 1996-12

**Authors:** G. S. Smith, C. Rieckenberg, W. E. Longo, D. L. Kaminski, J. E. Mazuski, Y. Deshpande, T. A. Miller

**Affiliations:** 1 Department of Surgery Saint Louis University School of Medicine Health Sciences Center 3635 Vista Avenue at Grand Boulevard St Louis Missouri 63110-0250 USA

## Abstract

We investigated whether an interleukin 1 receptor antagonist (IL-1ra) altered cellular release of prostanoids and leukotrienes in a transformed colonic cell line (CACO-2) in the presence of proinflammatory stimuli. Cellular inflammation was induced by treatment with lipopolysaccharide (LPS) or the cytokine, interleukin 1 beta (IL-1_β_). In a separate set of experiments, cells were pretreated with IL-1ra prior to exposure to LPS or IL-1_β_. Prostaglandin E_2_ and leukotriene B_4_ (LTB_4_) levels were quantified by ELISA assays. Both LPS and IL-1_β_ exposure were noted to stimulate cellular PGE_2_ release, a response which was significantly inhibited by IL-1ra treatment. Either stimulant when administered alone failed to stimulate release of LTB_4_. When administered after IL-1ra pretreatment however, both stimuli caused a significant increase in LTB_4_ release. These results suggest that a cytokine receptor antagonist can selectively influence eicosanoid production in this cell line. Furthermore, this study suggests that a IL-1ra may have a future clinical role in the treatment of inflammatory disorders of the colon which are intimately linked to enhanced eicosanoid synthesis.


Mediators of Inflammation, 5, 449–452 (1996)

### The effect of an interleukin receptor antagonist (IL-1ra) on colonocyte eicosanoid release

G. S. Smith, C. Rieckenberg, W. E. Longo,[CA]
D. L. Kaminski, J. E. Mazuski, Y. Deshpande and T. A. Miller

Department of Surgery, Saint Louis University School of Medicine, Health Sciences Center, 3635 Vista Avenue at Grand Boulevard, St Louis, Missouri 63110-0250, USA

[CA]Corresponding Author
Fax: (+1) 314 771 1945

**We investigated whether an interleukin 1 receptor antagonist (IL-1ra) altered cellular release of prostanoids and leukotrienes in a transformed colonic cell line (CACO-2) in the presence of proinflammatory stimuli. Cellular inflammation was induced by treatment with lipopolysaccharide (LPS) or the cytokine, interleukin 1 beta (IL-$1_\beta$). In a separate set of experiments, cells were pretreated with IL-1ra prior to exposure to LPS or IL-$1_\beta$. Prostaglandin $E_2$ and leukotriene $B_4$ ($LTB_4$) levels were quantified by ELISA assays. Both LPS and IL-$1_\beta$ exposure were noted to stimulate cellular $PGE_2$ release, a response which was significantly inhibited by IL-1ra treatment. Either stimulant when administered alone failed to stimulate release of $LTB_4$. When administered after IL-1ra pretreatment however, both stimuli caused a significant increase in $LTB_4$ release. These results suggest that a cytokine receptor antagonist can selectively influence eicosanoid production in this cell line. Furthermore, this study suggests that a IL-1ra may have a future clinical role in the treatment of inflammatory disorders of the colon which are intimately linked to enhanced eicosanoid synthesis.**

**Key words:** CACO-2, Colonocytes, Cytokines, Eicosanoids, Inflammation, Leukotrienes, Prostaglandins

### Introduction

Within the past two decades a large body of scientific information has surfaced regarding the molecular signals which control inflammation at both the local and systematic levels. Many inflammatory mediators have been recognized, including lipid-derived substances such as arachidonic acid metabolites, peptide regulatory molecules, and certain pro-inflammatory cytokines. A number of recent investigations of inflammatory bowel disease have focused on the role that these inflammatory mediators may play in initiating and perpetuating disease processes. Epithelial cells have been shown to produce certain cytokines under specific circumstances and recent investigation has focused on the role that various humoral mediators of inflammation including: autacoids such as platelet activating factor (PAF), eicosanoids, and cytokines play in the initiation and maintenance of intestinal inflammatory states.[1–3] Cytokines are known to be important in mediating many aspects of both inflammatory and immunologic responses, and it is likely that they play a similar role in initiating intestinal injury observed with inflammatory bowel disease. Similarly, LPS is known to contribute to the local inflammation and cellular toxicity associated with Gram negative infections[4] and is itself known to lead to enhanced expression of a variety of cytokines that orchestrate inflammation, immune responses,[5,6] and the release of arachidonic acid metabolites.[7]

IL-1ra is a natural monocyte protein (22KD) that has been cloned and expressed in recombinant form.[8,9] It has become a useful probe to investigate the role of IL-$1_\beta$ in the pathogenesis of inflammatory disease states. The aim of the present study was to determine the effect of IL-1ra upon cellular release of eicosanoids in colonocytes exposed to known pro-inflammatory stimuli.

### Materials and Methods

A transformed colonocyte cell line CACO-2 (American Type Culture Collection, Rockville, MD), was utilized in all experiments employing culture techniques described previously.[10] Briefly, the cells were maintained in Eagle's minimum essential medium (MEM) supplemented with 20% fetal bovine serum containing gentamicin (0.1 mg/ml) and 1% non-essential

© 1996 Rapid Science Publishers amino acids (Sigma, St Louis, MO). The cells were grown in a 37°C, humidified, 5% $CO_2$ incubator, and maintained by weekly passage. The cells were plated in six-well tissue culture plates (Costar, Cambridge, MA) with an initial density of $2 \times 10^5$ cells per well and allowed to grow to confluence. All experiments were conducted on cells which were 1–3 days post-confluent. The viability of the cells was assessed by trypan blue exclusion and morphology was evaluated by routine phase contrast microscopy.

Prior to the experiments, the cells were incubated for 24 h in serum free media. At the time of the experiments, the cells were washed twice with Krebs-Ringer bicarbonate buffer (KRB) and then the cells were exposed to lipopolysaccharide (10 μg/ml; Sigma Chemical, St Louis, MO) or IL-$1_\beta$ (0.1 μM; RND Systems, Minneapolis, MN) for 1 h. Previous studies in our own laboratory have demonstrated significant release of eicosanoids under such experimental conditions.[11,12] To evaluate the effects of a cytokine antagonist on such eicosanoid release, IL-1ra (100 μg/ml; Synergen Inc., Boulder, CO) was dissolved in 5% sodium carbonate solution and diluted in KRB buffer before being added to the cells for a 1 h preincubation period prior to subsequent exposure to LPS or IL-$1_\beta$. Control wells were similarly treated with 1 ml of fresh media for a similar time period. At the conclusion of the experiments, the media was collected for eicosanoid determinations and the plates frozen at −80°C until quantitative analysis of proteins was performed.

To determine protein concentrations, 0.3 ml of 1 N sodium hydroxide was added to each well and the wells were then thoroughly scraped. The resultant supernatants were collected and incubated in this solution for 1 h at 37°C. Protein levels were then determined with a BCA protein kit (Pierce Chemicals, Rockford, IL) using a microtitre plate protocol and serial dilutions of bovine serum albumin as internal standards.[13] $PGE_2$ and leukotriene $B_4$ ($LTB_4$) assays were performed in duplicate in buffer solutions without separation by a competitive enzyme assay which utilizes an acetylcholinesterase tracer (Cayman, Ann Arbor, MI). The eicosanoid concentrations were determined by spectrophotometric analysis of all samples after addition of Ellman's reagent and compared with standard curves generated under identical conditions. The data are presented as mean eicosanoid release (pg/mg protein) ± S.E.M. Statistical analysis was performed by analysis of variance with a Scheffe's *post hoc* test. Significance indicates a $p$ value $< 0.05$.

### Results

As indicated in Table 1, unstimulated CACO-2 cells released basal levels of $PGE_2$ and $LTB_4$ into the media. Incubation of these same cells with LPS and IL-$1_\beta$ significantly increased the release of $PGE_2$, although $LTB_4$ levels remained equivalent to the basal state. Similarly, treatment of these cells with IL-1ra alone increased the basal release of $PGE_2$ but had no effect on $LTB_4$ levels. Interestingly, IL-1ra treatment in the presence of LPS or IL-$1_\beta$ reversed the noteworthy increases in $PGE_2$ release and led to a paradoxical increase in cellular release of $LTB_4$.

### Discussion

The potential role that various cytokines play in the pathogenesis of inflammatory bowel disease has generated increased interest. In two models of experimental colitis[14,15] inflamed colonic mucosae were found to produce and release significantly higher amounts of IL-1 compared with normal colonic mucosae. Increased production of IL-1 as measured by bioassays and by ELISA has been reported during active disease states in both ulcerative colitis and Crohn's disease.[16,17] In support of these observations, Satsangi *et al.*[18] reported that peripheral blood mononuclear cells obtained from patients with active Crohn's disease produced significantly higher quantities of IL-1 under *in vitro* conditions when compared with normal control cells.

The relationship between the cytokine system and arachidonic acid pathways is complex. A variety of cellular and humoral mediators (i.e. pro-inflammatory eicosanoids) may be responsible for the mucosal damage observed in colonic inflammation. Although some cytokines are known to directly damage cells, or to augment the cytotoxicity of various leukocytes, some are known to directly stimulate the synthesis of

**Table 1.** The effect of LPS (10 μg/ml), IL-$1_\beta$ (0.1 μM) and IL-1ra (100 μg/ml) on cellular $PGE_2$ and $LTB_4$ release

| | $PGE_2$ (pg/mg protein) | $LTB_4$ (pg/mg protein) |
|---|---|---|
| Untreated | 97.4 ± 7.6 | 0.14 ± 0.03 |
| LPS | 510.5 ± 173.1 | 0.13 ± 0.01 |
| IL-$1_\beta$ | 612.0 ± 111.3* | 0.11 ± 0.01 |
| IL-1ra | 196.5 ± 51.4* | 0.18 ± 0.02 |
| LPS + IL-1ra | 114.0 ± 25.1** | 0.43 ± 0.09** |
| IL-$1_\beta$ + IL-1ra | 102.1 ± 33.9† | 0.41 ± 0.03† |

*Note:* Each value represents the mean ± S.E.M. of six values from 6 wells containing $2 \times 10^5$ CACO-2 cells.
*Significant from untreated cells; **significant from LPS; †significant from IL-$1_\beta$.

pro-inflammatory eicosanoids. Under these conditions, cell membrane 1-alkyl-2-arachidonyl-1-SN-glycerol-3-phosphocholine (G3PC) is acted upon by phospholipase $A_2$ to form arachidonic acid and 1 alkyl-glycerol-phosphocholine (1 alkyl-GPC). Arachidonic acid in turn can be converted into eicosanoids by lipoxygenase and cyclooxygenase pathways, thus linking interleukin production with the synthesis of eicosanoids during inflammatory states.[19]

Our results indicate that CACO-2 cells release significant amounts of $PGE_2$ in response to stimulation with either LPS or $IL\text{-}1_\beta$, suggesting that these stimulants have the capacity to directly regulate the synthesis of prostanoids in these cells. Since $LTB_4$ levels were not enhanced under these same conditions, these data further suggest that either the lipoxygenase arm of the arachidonic acid cascade does not respond in a similar manner to these same agonists, or more likely that the noted increases in $PGE_2$ can exhibit an inhibitory effect on $LTB_4$ formation in these cells. The latter concept is supported at least in part, by the work of Ham *et al.*,[20] who reported that $PGE_2$ directly inhibited the release of $LTB_4$ from activated neutrophils.

Our results also indicate that treatment of CACO-2 cells with a selective IL-1 receptor antagonist (IL-1ra) induced a significant increase in cellular release of $PGE_2$, suggesting a similar stimulatory effect on prostanoid synthesis in this cell line. Although an apparent explanation for these effects is lacking, such stimulation could occur if the IL-1ra had a direct biological effect on the prostaglandin cascade or if it could induce the synthesis of yet another cytokine whose effects would be mediated through endogenous prostaglandin synthesis. In support of this contention is the work of Oshima *et al.*,[21] who reported that in rats injected systemically with IL-1ra, there was a significant elevation in gastric mucosal $PGE_2$ compared with control treated animals.

An interesting observation in the present study was the noted effect of IL-1ra pretreatment upon $PGE_2$ and $LTB_4$ levels in cells that were stimulated with either LPS or $IL\text{-}1_\beta$. Under these conditions $PGE_2$ levels were noted to return to baseline and $LTB_4$ levels were noted to be significantly elevated, representing a paradoxical shift in arachidonic acid metabolism. Although not obvious, a potential explanation for the observed results could be an apparent relationship between regulatory mechanisms governing leukotriene synthesis directly by one or more prostaglandins which is intimately orchestrated by IL-1ra. Under these conditions, $PGE_2$ when available in sufficient amounts could exert an inhibitory effect on $LTB_4$ synthesis and release. If this were the case, then decreases in or inhibition of $PGE_2$ synthesis would obviate this inhibitory effect and result in enhanced $LTB_4$ as detected in this study. In support of this contention are numerous studies which demonstrate such effects in adjuvant arthritis.[22–24]

Thus, there is ample evidence to implicate various eicosanoids, and proinflammatory cytokines in the pathogenesis of colitis. Although most of the production of these mediators has been attributed to the inflammatory cells present in bowel wall, there is also increasing evidence for the production of some of these potentially proinflammatory compounds by the mucosal epithelial cells themselves. Thus, it is possible that epithelial cells may actually contribute significantly to the pathological process in patients with inflammatory bowel disease. Since this process has not yet been thoroughly delineated, further attempts to better characterize epithelial cell responses to initiators of mucosal inflammation, particularly with regard to the release of different types of prostanoids are warranted. A better understanding of the mechanism(s) by which the local inflammatory process in the bowel is generalized to produce these systemic signs and symptoms may further suggest new therapeutic options which could offer some palliation to these patients.

### References

1. Campbell CA, Walker-Smith JA, Hindocha P, Adinolfi M. Acute phase proteins in chronic inflammatory bowel disease in childhood. *J Ped Gastroenterol Nutr* 1982; 1: 193–200.
2. Chambers RE, Stross P, Barry RE, Whicher JT. Serum amyloid A protein compared with C-reactive protein, alpha 1-antitrypsin and alpha 1-acid glycoprotein as a monitor of inflammatory bowel disease. *Eur J Clin Invest* 1987; 17: 460–467.
3. Prantera C, Davoli M, Lorenzetti R, Pallone F, *et al.* Clinical and laboratory indicators of extent of ulcerative colitis. Serum C-reactive protein helps the most. *J Clin Gastroenterol* 1988; **10**: 41–45.
4. Cubulsky NME, Chan MK, Movat HZ. Acute inflammation and microthrombosis induced by endotoxin, IL-1 and TNF and their implication in gram-negative infection. *Lab Investigation* 1988; **55**: 365–378.
5. Uhich TR, DelCastillo J, Keys M, Granger GA, Ni RX. Kinetics and mechanisms of recombinant human IL-1 and TNFα induced changes in circulating numbers of neutrophils and lymphocytes. *J Immun* 1987; 139: 3406–3415.
6. Dinarello CA, Cannon JG, Wolff SM, Bemheim HA, Buttler B, Cerami A, Figari IS, Palladino MA, O'Conner JV. TNF is an endogenous pyrogen and induces production of IL-1. *J Exp Med* 1986; **163**: 11433–11450.
7. Sirko S, Bishai I, Coceani F. Prostaglandin formation in the hypothalamus *in vivo*: effect of pyrogens. *Am J Pathol* 1989; **256**: R616–R624.
8. Eisenberg SP, Evans RJ, Arend WP, Verderber E, Brewer MT, Hannum CH, Thompson RC. Primary structure and functional expression from complementary CDNA of a human IL-1 receptor antagonist. *Nature* 1990; **343**: 336–340.
9. Hannum CH, Wilcox CJ, Arend WP, Joslin FG, Dripps DJ, Heimdal PL, Armes LG, Sommer A, Eisenberg SP, Thompson RC. IL-1 receptor antagonist activity of a human IL-1 inhibitor. *Nature* 1990; **85**: 1694–1697.
10. Stratton MD, Chandel B, Deshpande Y, Kaminski DL, Li AP, Vernava AM, Longo WE. The effect of clostridium difficile toxin on colonocyte prostanoid production. *Prostaglandins* 1994; **48**: 367.
11. Kaminski DL, Amir G, Deshpande Y, Beck D, Li AP. Studies on the etiology of acute acalculous cholecystitis: the effect of lipopolysaccharide of human gallbladder mucosal cells. *Prostaglandins* 1994; **47**: 319–330.
12. Kaminski DL, Feinstein WK, Deshpande YG. The production of experimental cholecystitis by endotoxin. *Prostaglandins* 1994; **47**: 233–237.
13. Wiechelman K, Braun R, Fitzpatric J. Investigation of the bicinchoninic acid protein assay; identification of the groups responsible for color formation. *Anal Biochem* 1988; **175**: 231–237.
14. Rachmilewitz D, Simon PL, Schwartz LW, Griswold DE, Fondacaro JD, Wasserman MA. Inflammatory mediators of experimental colitis in rats. *Gastroenterology* 1989; **97**: 326–337.
15. Rachmilewitz D, Simon PL, Sjogrn R, Fondacaro JD, Wasserman MA, Dodecker E. Investigation of IL-1 activity in the intestinal mucosa of Crohn's disease (CD) and ulcerative colitis patients. *Gastroenterology* 1989; **94**: A363.
16. Durum SK, Schmidt JA, Oppenheim JJ. Interleukin 1: an immunological perspective. *Annu Rev Immunol* 1985; **2**: 263–287.
17. Elner VM, Streiter RM, Elner SG, Baggiolini M, Lindley I, Kunkel SL. Neutrophil chemotactic factor (IL8) gene expression by cytokine-treated retinal pigment epithelial cells. *Am J Pathol* 1990; **136**: 745–750.
18. Satsangi J, Wolstencroft RA, Cason J, Ainley CC, Dumonde CC, Thompson RPH. Interleukin 1 in Crohn's disease. *Clin Exp Immunol* 1987; **67**: 594–605.
19. Ulich TR, Busser K, Longmuir KJ. Cytokine and calcium ionophore A23187—mediated arachidonic acid metabolism in neutrophils. *Cytokine* 1990; **2**: 280–286.
20. Ham EA, Soderman DD, Zanetti ME, Dougherty HW, McCauley E, Kuehl Jr FA. Inhibition by prostaglandins of leukotriene $B_4$ release from activated neutrophils. *Proc Natl Acad Sci USA* 1983; **80**: 4349–4353.
21. Oshima A, Wakabayashi G, Shimagu M, Yoshida A, Iwata K, Kitajima M. *Gastroenterology* 1995; **108**: A184.
22. Asprinall RL, Cammarata PS. Effect of prostaglandin $E_2$ on adjuvant arthritis. *Nature (London)* 1969; **224**: 1320.
23. Zurier RB, Quagliata F. Effect of prostaglandin $E_1$ on adjuvant arthritis. *Nature (London)* 1971; 304–305.
24. Zurier RB, Hoffstein S, Weissmann G. Suppression of active and chronic inflammation in adrenalectomized rats by pharmacologic amounts of prostaglandins. *Arthritis Rheum* 1973; **16**: 606–618.

**Received 22 July 1996;**
**accepted in revised form 27 September 1996**
